# Chemopreventive apigenin controls UVB-induced cutaneous proliferation and angiogenesis through HuR and thrombospondin-1

**DOI:** 10.18632/oncotarget.2551

**Published:** 2014-10-15

**Authors:** Xin Tong, Salida Mirzoeva, Dorina Veliceasa, Bryan B. Bridgeman, Philip Fitchev, Mona L. Cornwell, Susan E. Crawford, Jill C. Pelling, Olga V. Volpert

**Affiliations:** ^1^ Department of Pathology, Northwestern University Feinberg School of Medicine, Chicago, IL, USA; ^2^ Department of Radiology, Northwestern University Feinberg School of Medicine, Chicago, IL, USA; ^3^ Department of Urology, Northwestern University Feinberg School of Medicine, Chicago, IL, USA; ^4^ Department of Pathology, Saint Louis University School of Medicine, St Louis, MO, USA; ^5^ Robert H. Lurie Comprehensive Cancer Center, Northwestern University Feinberg School of Medicine, Chicago, IL, USA

## Abstract

Plant flavonoid apigenin prevents and inhibits UVB-induced carcinogenesis in the skin and has strong anti-proliferative and anti-angiogenic properties. Here we identify mechanisms, by which apigenin controls these oncogenic events. We show that apigenin acts, at least in part, via endogenous angiogenesis inhibitor, thrombospondin-1 (TSP1). TSP1 expression by the epidermal keratinocytes is potently inhibited by UVB. It inhibits cutaneous angiogenesis and UVB-induced carcinogenesis. We show that apigenin restores TSP1 in epidermal keratinocytes subjected to UVB and normalizes proliferation and angiogenesis in UVB-exposed skin. Importantly, reconstituting TSP1 anti-angiogenic function in UVB-irradiated skin with a short bioactive peptide mimetic representing exclusively its anti-angiogenic domain reproduced the anti-proliferative and anti-angiogenic effects of apigenin. Cox-2 and HIF-1α are important mediators of angiogenesis. Both apigenin and TSP1 peptide mimetic attenuated their induction by UVB. Finally we identified the molecular mechanism, whereby apigenin did not affect TSP1 mRNA, but increased *de novo* protein synthesis. Knockdown studies implicated the RNA-binding protein HuR, which controls mRNA stability and translation. Apigenin increased HuR cytoplasmic localization and physical association with TSP1 mRNA causing *de novo* TSP1 synthesis. HuR cytoplasmic localization was, in turn, dependent on CHK2 kinase. Together, our data provide a new mechanism, by which apigenin controls UVB-induced carcinogenesis.

## INTRODUCTION

Unlimited proliferation is one of the hallmarks of cancer [1, 2]; however, to realize their capacity for unlimited growth *in vivo* tumors overcome multiple constraints posed by normal host tissues. To grow beyond the threshold size of several mm in diameter, tumors require autonomous blood supply, which is generated through neovascularization (angiogenesis), another hallmark of cancer [2]. Endogenous anti-angiogenic proteins are present at high levels in normal adult tissues and their expression is often decreased in the course of tumor progression, an event permissive for tumor angiogenesis [3]. Thrombospondin-1 (TSP1) was the first endogenous angiogenesis inhibitor to be identified [4] and its loss is among the critical events in progression of multiple cancers including carcinomas of the breast and colon as well as skin cancers [5, 6]. Previous studies identified TSP1 as a critical angiogenesis inhibitor in human and mouse skin, whose expression is blocked by ultraviolet B (UVB) [7, 8]. Ectopic TSP1 delays the growth of xenografted cutaneous tumors in mice and TSP1 transgene mitigates acute and long-term UVB damage in the skin [9, 10]. Thus TSP1 is an important therapy target in multiple cancer types, including non-melanoma skin cancers and melanoma [11, 12]. Here we report that plant flavonoid apigenin effectively restores TSP1 expression in UVB-irradiated skin.

Apigenin, is a potent chemopreventive agent and inhibitor of UVB-induced skin carcinogenesis [13]. We have shown that apigenin causes G1 and G2/M growth arrest by targeting cyclins B1 and D1 [14, 15] and by promoting p53-dependent transcription [16] in epidermal keratinocytes exposed to UVB. In colon and prostate cancers, apigenin causes growth arrest and cell death via DR5 [17, 18] and E-cadherin [19]. Importantly, apigenin blocks cyclooxygenase (Cox)-2 and its target Prostaglandin E_2_ (PGE_2_) [20, 21] via complex regulatory mechanism, whereby two RNA-binding protein HuR and translational repressor T-cell specific antigen 1-related protein inhibit Cox-2 induction by UVB [22].

In addition to its cytotoxic and cytostatic effects, apigenin inhibits angiogenesis. In non-skin tissues, this has been attributed to the suppression HIF-1α and VEGF [23–26]. Other *in vitro* studies indicate the role of nitric oxide (NO) and IL6/STAT pathways [27, 28]. Here, we show that apigenin inhibits cutaneous angiogenesis, at least in part, by maintaining high levels of anti-angiogenic TSP1. Apigenin mitigated the TSP1 loss due to UVB exposure in the epidermal keratinocytes in culture and in mouse skin. Moreover, it alleviated angiogenic and proliferative responses to UVB radiation in the skin *in vivo*. The regulation of TSP1 by apigenin was post-transcriptional. TSP1 mRNA was strongly decreased in UVB-treated cells and remained low in the presence of apigenin. In contrast, apigenin increased translational activity of existing TSP1 transcripts. Specifically, it increased cytosolic localization of the RNA-binding protein Human antigen R (HuR) and HuR association with TSP1 mRNA, causing enhanced translation and high TSP1 protein levels.

Thrombospondin-1 is a large multi-functional protein whose anti-angiogenic activity was mapped to the Type 1 malarial repeats, with CD36 as one of the signaling receptors on the vascular endothelium (reviewed in [29]). Importantly, peptide mimetic of TSP1, ABT-898, which represents anti-angiogenic thrombospondin repeats (TSR2) and blocks angiogenesis in CD36-dependent manner [29], was sufficient to reproduce the anti-angiogenic and cytostatic effects of apigenin in the skin suggesting a key functional role of TSP1 in apigenin's chemoprevention of UVB-induced oncogenic events. To our knowledge, this is the first demonstration of the regulation of an endogenous anti-angiogenic factor by apigenin, with dramatic effect on cutaneous angiogenesis.

## RESULTS

### Apigenin restores TSP1 expression in UVB-treated skin keratinocytes

**Figure 1:**
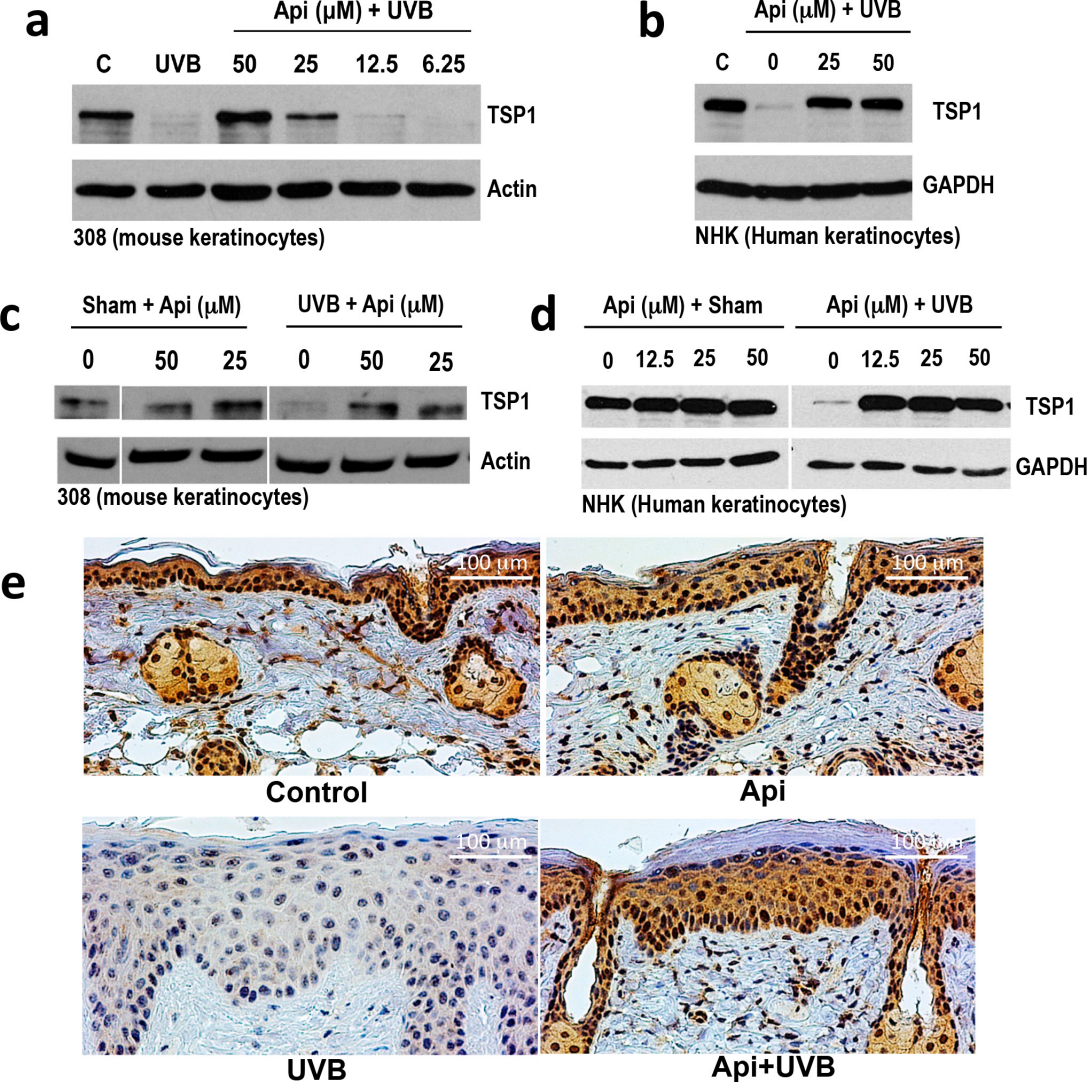
The effect of UVB and apigenin on TSP1 expression in epidermal keratinocytes *in vitro* and *in vivo* **(a, b)** Mouse 308 keratinocytes (a) and normal human epidermal keratinocytes (NHEKs, b) were pre-treated with indicated concentrations of apigenin (Api) for 1 hr before UVB exposure (1000J/m^2^), and TSP1 was detected by Western Blot in cell extracts collected 12 h after UVB irradiation. **(c)** 308 cells were subjected to sham or UVB irradiation and apigenin added to cell cultures at indicated concentrations immediately after irradiation. The extracts were harvested after additional 12 hrs and analyzed by Western blot. **(d)** NHEKs were pre-treated with increasing concentrations of apigenin, where indicated and subjected to sham or UVB irradiation. Cell extracts were collected 16 hr post-irradiation and TSP1 expression analyzed by Western blot. Actin and GAPDH were used to assess loading. **(e)** SKH-1 mice were treated with UVB (5 consecutive days, 1300 J/m^2^ /day). Where indicated, the animals were pre-treated with topical apigenin (5 μMol in DMSO/acetone). Dorsal epidermis was harvested, sectioned and stained for TSP1.

Seeking mechanisms underlying apigenin's effects in the skin, we assessed TSP1 levels in mouse and human keratinocytes exposed to UVB and treated with apigenin. In immortalized 308 mouse keratinocytes (Fig. 1a) and in primary normal human epidermal keratinocytes (NHEKs, Fig. 1b), TSP1 expression was dramatically decreased 12 hr following UVB exposure. Importantly, both pre-treatment with apigenin (1-2 hrs prior to UVB) Fig. 1a, b and d) and treatment immediately after UVB irradiation (Fig. 1c) similarly increased TSP1 protein levels. In sham-irradiated cells, apigenin caused a moderate increase of already high TSP1 levels, compared to a robust increase in UVB-irradiated cells, where TSP1 was barely detectable (Fig. 1d).

Importantly, apigenin restored TSP1 expression in the UVB-treated skin *in vivo*. As was shown previously, TSP1 expression (detected by immunohistochemistry, IHC) was vanishingly low in the skins of mice after UVB irradiation. In contrast, in animals treated with topical apigenin, TSP1 staining in the epidermis was similar to that of untreated controls (Fig. 1e). Interestingly, one of the TSP1 anti-angiogenic receptors, CD36, was similarly downregulated by UVB and restored by apigenin (Supplementary Fig. 1).

### Apigenin and TSP1 peptide mimetic blocks UVB-induced epidermal thickening

**Figure 2:**
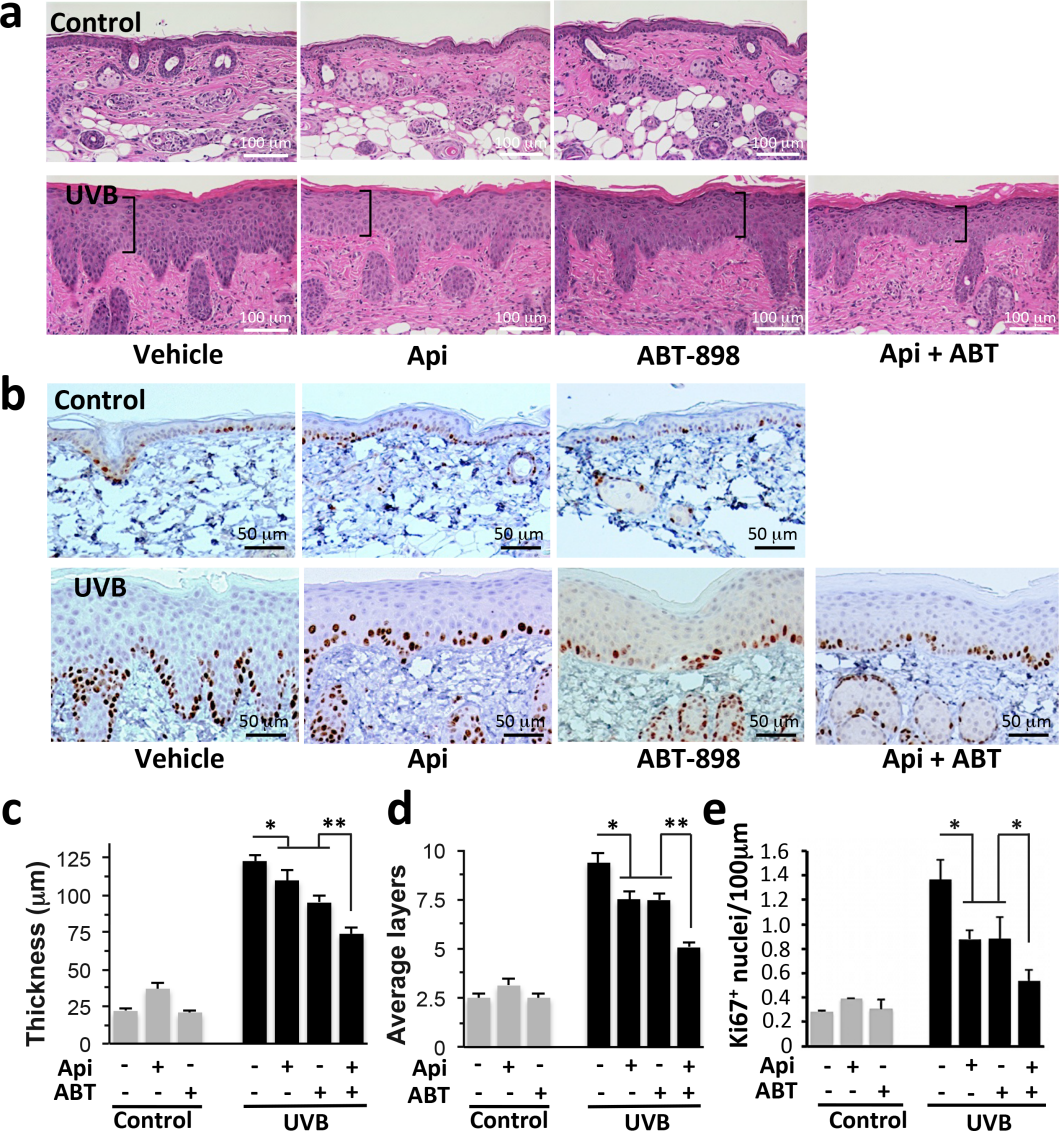
The effect of apigenin and TSP1 peptide mimetic (ABT-898) on UVB-induced skin thickening and proliferation Mice were subjected to sham or UVB radiation (1300 J/m^2^ daily, 5 days). Where indicated, topical Apigenin (5 μMol) and subcutaneous ABT-898 (100 ng/ml in PBS, 30 mg/kg) were given 2 h prior to each UVB exposure. Mice were sacrificed at 12, 24 and 48 h after final exposure and dorsal skins harvested, fixed in formalin and paraffin-embedded. Five μm sections were stained with Hematoxylin and Eosin (H&E) for morphometric analysis (see Methods). **(a, c, d)** Dorsal skins harvested at 12 hrs post irradiation were used for H&E staining and subsequent morphometry analysis. Note the visibly decreased thickness of the UVB-irradiated epidermis in mice treated with apigenin and/or ABT-898. **(b, e)** IHC for proliferation marker Ki-67 at 24 hrs post irradiation. **(c)** Measurement of epidermal thickness. At least 3 sections per animal and 3 animals per data point were evaluated. **(d)** The number of epidermal cell layers was determined in 3 random measurements per section, a minimum of 2 sections per animal, in 3 animals per data point. **P value <0.0001; *P <0.003 as determined by one-way ANOVA. **(e)** Quantitative analysis of the experiment in **(b)** was performed using ImageJ64 software on thresholded images, using “Particle Analysis” function. A minimum of three 10x fields from 3 independent sections were examined and Ki-67 positive nuclei calculated per linear 100 μm of epidermis. Hair follicles were not included in analysis. *P<0.01 as was determined by two-tailed Student's T-test using pairwise comparisons.

TSP1 active regions have been extensively analyzed and its anti-angiogenic activity mapped to the Type 1 malarial repeats (TSR), which yielded series of highly active TSP1 peptide mimetics. One of them, ABT-898, derived from TSR2, is especially potent in multiple models of angiogenesis and tumor growth and reproduces a full spectrum of TSP1 angioinhibitory effects [30–32]. We therefore tested its ability to reproduce apigenin's effects in the UVB-treated skin. Epidermal thickening was noted as early as 12 hrs after UVB exposure (Fig. 2a, c) and lasted up to 48 hours (Supplementary Fig. 2). The thickening was largely due to hyperproliferation, as was evidenced by increased number of the keratinocyte layers (Fig. 2a, d). Apigenin significantly decreased both the thickness and the number of layers in the epidermis of irradiated mice (Fig. 2a, c, d). Importantly, subcutaneous injections of ABT-898 had a similar effect and the combination of apigenin and ABT-898 caused even greater decrease in epidermal thickness and the number of keratinocyte layers (Fig. 2c, d). In agreement, UVB caused an approximately 4-fold increase in proliferation, as was measured by IHC for Ki-67 (Fig. 2b, e). Treatment with either apigenin or ABT-898 caused a 2-fold reduction in the number of Ki-67 positive nuclei per linear 100μm of epidermis. The combined effect of apigenin and ABT-898 exceeded the effects of each compound alone and this difference was statistically significant (Fig. 2e).

### ABT-898 recapitulates the anti-angiogenic effect of apigenin in the skin

**Figure 3:**
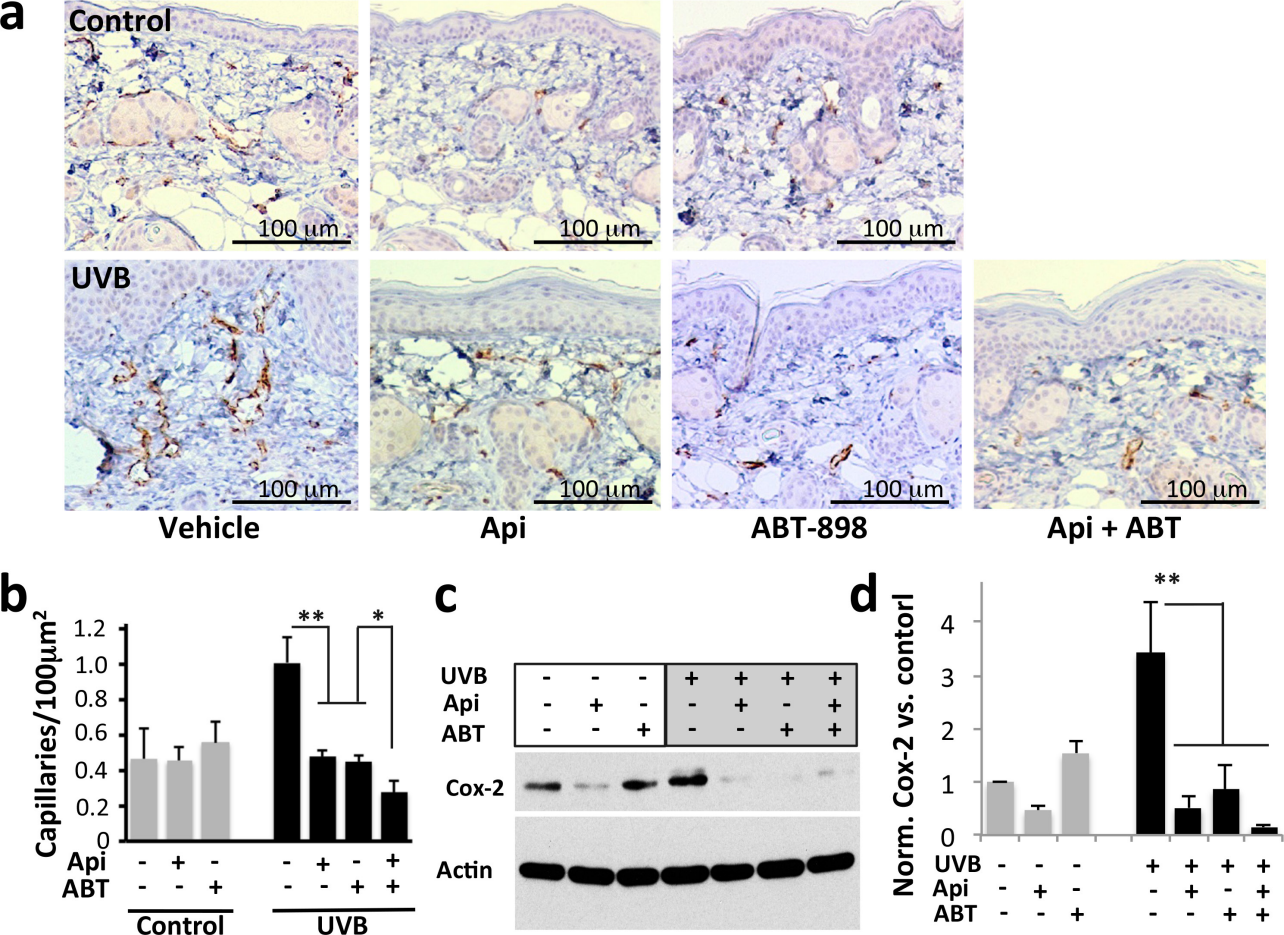
The effect of apigenin and ABT-898 on UVB-induced angiogenesis Mice were subjected to sham or UVB radiation (1300 J/m^2^ daily, 5 consecutive days). Topical Apigenin (5 μMol) and subcutaneous ABT-898 (100 ng/ml in PBS, 30 mg/kg) were given daily 1–2 h prior to UVB exposure. Mice were sacrificed 48 hr after final exposure and dorsal skins were harvested, formalin fixed and paraffin embedded. **(a)** IHC for an endothelial cell marker CD31. Note a visible increase in microvascular density (MVD) caused by UVB. **(b)** Quantitative analysis of the IHC was performed using “Object Count” function in ImageJ64 software. A minimum of 10x fields from 3 independent skin sections were examined and MVD calculated per 100 μm^2^ area; *P>0.1 **P<0.001 by two-tailed Student's T-test. **(c, d)** TSP1 and apigenin inhibit Cox-2 induction by UVB. Control and UVB-irradiated 308 keratinocytes were treated with apigenin (50μM) and ABT-898 (100nM) where indicated and Cox-2 in cell extracts was measured by Western blot (c) and quantified by densitometric analysis (ImageJ64 software) (d). The graph represents average of three independent experiments, all normalized to untreated control. **P<0.009

The anti-angiogenic effect of apigenin in the non-skin tissues has been shown previously [23, 33, 34] and attributed to the decreased production of VEGF-A, due to the blockade of hypoxic and growth factor signaling. However, apigenin's effect on cutaneous angiogenesis and TSP1 has not been documented. We therefore analyzed apigenin's anti-angiogenic effects in the skin and tested the ability of TSP1 anti-angiogenic peptide mimetic to reproduce these actions. UVB significantly increased microvascular density (MVD) in the dermis, as was measured using IHC for the endothelial marker, CD31 (Fig. 3a, b), and in the underlying adipose tissue (data not shown). In contrast, treatment with apigenin and/or ABT-898 inhibited UVB-induced angiogenesis by at least 60%, suggesting a critical role for TSP1 in the anti-angiogenic action of apigenin.

UVB is known to increase expression of Cox-2 [35, 36], a critical mediator of inflammation and angiogenesis [37, 38]. We have earlier shown that apigenin inhibits the induction of Cox-2 by UVB [20, 39]. ABT-898 alone caused a minor increase in CD31 staining (Fig. 3a, b) and Cox-2 expression (Fig. 3c, d), however this trend did not reach significance. In contrast, ABT-898 significantly reduced UVB-induced angiogenesis (Fig. 3b, P<0.001) and Cox-2 expression (Fig. 3c, d, P<0.009).

### ABT-898 blocks the stimulation of HIF pathway by UVB

Apigenin inhibits HIF pathway and VEGF production in non-skin tissues. We used ABT-898 to determine whether these effects of apigenin could be related to TSP1.

**Figure 4:**
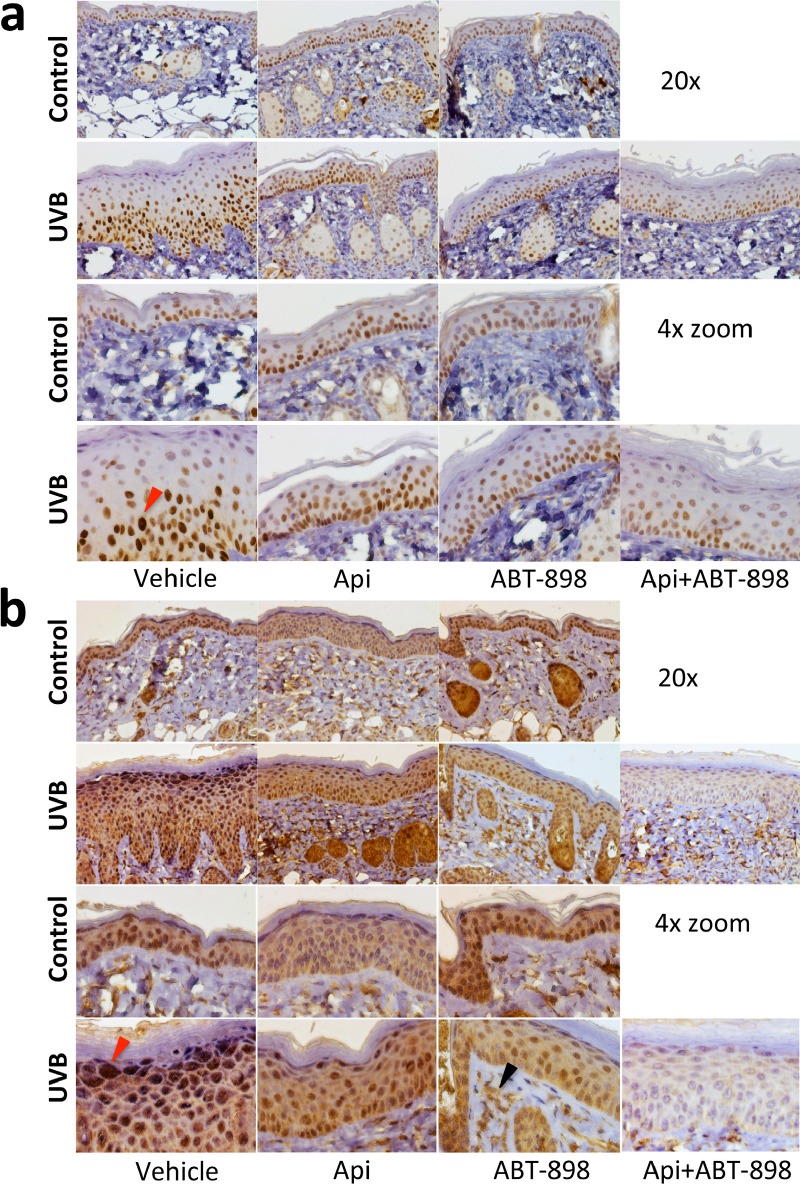
ABT-898 inhibits the induction of HIF pathway by UVB in epidermal keratinocytes Mice were subjected to sham or UVB radiation (1300 J/m^2^ daily, 5 days). Where indicated, topical Apigenin (5 μMol) and subcutaneous ABT-898 (100 ng/ml in PBS, 30 mg/kg) were given 2 h prior to each UVB exposure. Mice were sacrificed at 24 h after final exposure to UVB and dorsal skins harvested, fixed in formalin and paraffin-embedded. Five μm sections were stained with antibodes agains HIF-1α and VEGF (see Methods) and countesrtained with hematoxylin. **(a)** Staining for HIF-1α. Images were taken at 20x magnification. A 4x zoom is shown below. Red arrow points to the nucleus with increased HIF-1α after UVB irradiation. **(b)** VEGF stiaining. Note a significant increase in VEGF staining in the uppermost keratinocyte layers after UVB exposure (red arrow). Also note the lack of VEGF regulation by apigenin and ABT-898 in non-irradiated skin and visible decrease in VEGF staining in the skin that was exposed to UVB. Sub-epidermal VEGF staining (black arrow) is localized to intercellular spaces; it appears unaltered and serves as an internal control.

IHC for HIF-1α showed positive staining in control skin, predominantly in basal layer, which was not significantly altered by apigenin or ABT-898 (Fig. 4a). The expression of HIF-1α (staining intensity) increased 24 hrs post-irradiation (Fig. 4a, red arrow). This increase was abolished by apigenin and/or ABT-898 (Fig. 4a).

VEGF is the main pro-angiogenic target of the HIF pathway. Accordingly VEGF staining of epidermal keratinocytes was also increased by UVB 24 hrs post-irradiation (Fig. 4b, red arrow) and this increase was abolished by apigenin and by ABT-898, alone or in combination (Fig. 4b). In sub-epidermal skin layers, VEGF staining was localized mainly to the intercellular spaces (Fig. 4b, black arrow), presumably due to its binding to the extracellular matrix.

### Apigenin regulates TSP1 via the RNA-binding protein HuR

**Figure 5:**
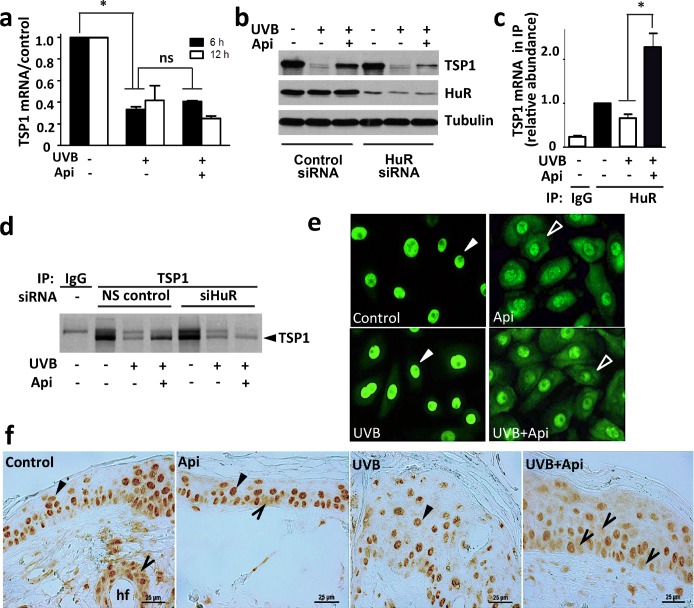
Apigenin regulates TSP1 translation via HuR **(a)** Apigenin did not restore TSP1 mRNA level in UVB-irradiated cells. Control and UVB-irradiated 308 mouse keratinocytes were treated with apigenin where indicated and TSP1 mRNA measured by real-time RT-PCR. Statistical significance was determined in pairwise comparison by two-tailed Student's T-test. *P<0.002; ns, not significant (P>0.4). **(b)** HuR was required for apigenin to maintain TSP1 expression. HuR knockdown in cells treated with control and HuR-specific siRNA was ascertained by Western blot (middle panel). The cells were then subjected to UVB irradiation and treated with apigenin or control vehicle. Note diminished TSP1 levels (top panel) upon HuR knockdown. The membrane was re-probed for alpha-tubulin to ascertain equal loading. **(c)** Apigenin increases HuR binding to TSP1 mRNA. The cells were treated with UVB and apigenin or control vehicle. The lysates were harvested and subjected to immunoprecipitation with HuR specific antibodies or control IgG and subsequent real-time PCR with primers for TSP1 mRNA. Note significant increase in the TSP1 mRNA in HuR protein complexes from the cells treated with the combination of UVB and apigenin. *P<0.008. **(d)** Apigenin controls *de novo* TSP1 protein synthesis via HuR. Mouse 308 keratinocytes were pre-incubated in methionine and cysteine-free medium and exposed to UVB in the absence and in the presence of apigenin. After 12 h the cells were given 15-min pulse of [^35^S] L-methionine and [^35^S] L-cysteine. The lysates were subjected to IP with anti-TSP1 antibody or normal mouse IgG (control). **(e, f)** Apigenin promotes cytoplasmic localization of HuR in UVB-irradiated keratinocytes and in the skin. **(e)** The cells were treated as indicated and HuR localization assessed by immunofluorescence (green). **(f)** Mice were subjected to UVB irradiation, with and without apigenin pre-treatment as above. Dorsal skins were harvested, sectioned and HuR localization assessed by IHC. Note nuclear HuR localization (filled arrows) in the absence of apigenin and cytoplasmic presence of HuR (empty arrowheads) in apigenin-treated cells/skins. Note a more pronounced cytoplasmic HuR localization in skins treated with UVB and apigenin combination.

Pre-treatment with apigenin strongly increased TSP1 levels in the UVB-irradiated skin and epidermal keratinocytes (Fig. 1). Surprisingly, apigenin failed to restore TSP1 mRNA levels in UVB-irradiated keratinocytes (Fig. 5a), suggesting regulation at post-transcriptional level. We therefore investigated its potential regulatory effect on TSP1 translation. We focused on ELAV-like 1 human antigen R (HuR) because it is one of apigenin's molecular targets [20] and is involved in control of mRNA stability and translation. HuR can shift the ratio between translated and untranslated mRNA towards the active fraction [40] and has been reported to promote *de novo* TSP1 synthesis [41]. We used siRNA knockdown to test the role of HuR role in TSP1 regulation by apigenin. Indeed, HuR silencing diminished the ability of apigenin to rescue TSP1 expression in UVB-treated cells (Fig. 5b). Moreover, real time RT-PCR of mRNA from HuR complexes isolated by immunoprecipitation, showed increased recruitment of TSP1 mRNA in apigenin-treated keratinocytes (Fig. 5c). Importantly, metabolic labeling with [^35^S]-methionine showed higher *de novo* TSP1 synthesis in UVB irradiated cells treated with apigenin, which was abolished in proportion with the extent of HuR knockdown (Fig. 5d). Analysis of whole cell extracts was performed to show that equal amount of [^35^S]methionine/cysteine was incorporated into the cells and that protein translation in general was not affected by the different treatments.

### Apigenin regulates HuR subcellular localization

In the cell, HuR activity is determined by its localization. Inactive HuR is sequestered in the nuclei and cytoplasmic HuR becomes active and controls mRNA stability and translation [42]. In sham- and UVB-irradiated 308 keratinocytes, apigenin treatment significantly increased the levels of HuR in the cytoplasm 4 hrs post irradiation (Fig. 5e). Importantly, apigenin increased cytoplasmic HuR in UV-irradiated mouse skin *in vivo* 24 hours post irradiation (Fig. 5f).

**Figure 6:**
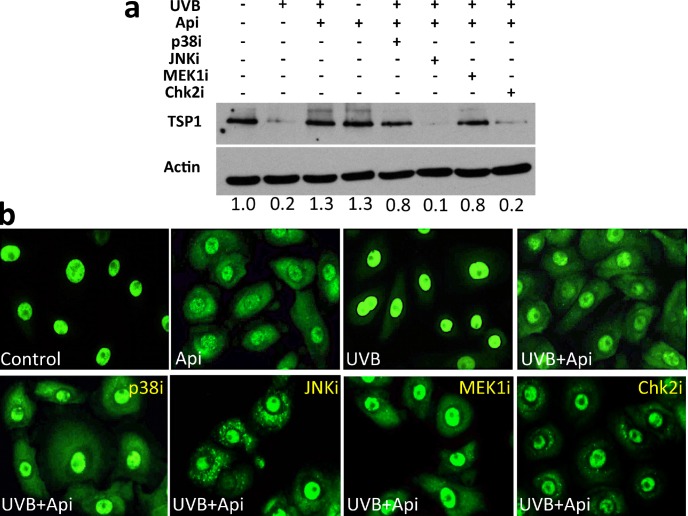
HuR-translocation and recovery of TSP1 expression by apigenin requires Chk2 kinase NHEKs were treated with UVB and apigenin. Specific inhibitors of p38 (SB203580), MEK1 in the Erk1/2 pathway (U0126) and Chk2 inhibitor II, were added at 10 μM and JNK-1 inhibitor SP600125, was added at 50 μM. **(a)** TSP1 in whole cell extracts was measured by Western blot. Note that apigenin failed to restore TSP1 expression in the presence of JNK-1 and Chk-1 inhibitors. Numbers under the panel indicate values determined by densitometry (ImageJ) adjusted for loading (Actin) and normalized to untreated control. **(b)** NHEKs grown on coverslips were fixed and immunofluorescence performed to visualize HuR. Note decreased cytoplasmic staining in the presence of Chk2 inhibitor.

HuR localization can be controlled by multiple kinases including Erk1/2, p38, JNK-1 and Chk2 (reviewed in [43]). All of these are perturbed by apigenin in diverse cell types [44–46]. To identify the player(s) involved in apigenin regulation of HuR in the skin, we have measured TSP1 expression and assessed HuR cytoplasmic localization in the NHEK cells pre-treated with specific kinase inhibitors. The ability of apigenin to restore TSP1 expression in the UVB-irradiated keratinocytes was impaired when JNK-1 and Chk2 were blocked (Fig. 6a). Chk2 inhibitor caused a marked decrease in HuR cytoplasmic localization (Fig. 6b) suggesting that Chk2 is a critical mediator of apigenin's regulation of HuR and TSP1. In addition, JNK-1 inhibitor markedly altered HuR staining in the cytoplasm, with striking punctate pattern. A similar, although less pronounced pattern was observed in the presence of Chk2 inhibitor (Fig. 6b).

However, JNK-1 blockade, but not Chk2 blockade, decreased TSP-1 levels regardless of apigenin and/or UVB exposure. (Supplementary Figure 3).

**Figure 7:**
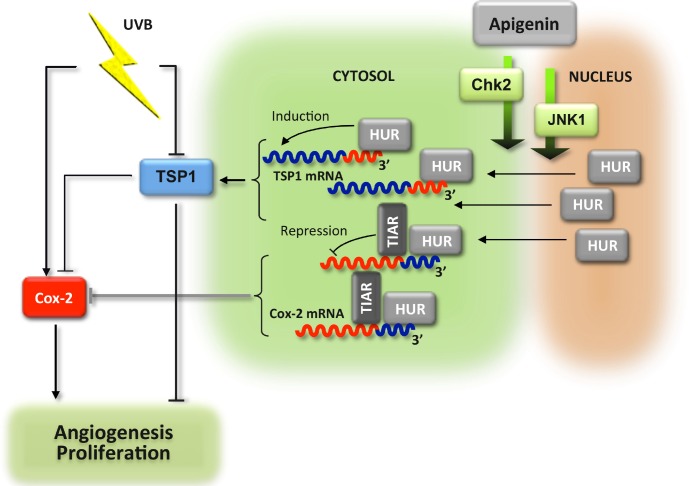
Schema of the anti-angiogenic cascade induced by apigenin in the UVB-irradiated skin UVB exposure increases proliferation of the epidermal keratinocytes and elevated angiogenesis in sub-epidermal layers. These events are dependent on the increased production of Cox-2 and reduced levels of mRNA encoding anti-angiogenic TSP1. In the presence of apigenin, HuR enters cytoplasm of the epidermal keratinocytes where it binds and stabilizes TSP1 mRNA and increases *de novo* translation. In contrast, Cox-2 mRNA becomes destabilized. The accumulation of TSP1 protein results in attenuated proliferation and angiogenesis. In addition, TSP1 decreases Cox-2 production via unknown mechanism, and further augments anti-proliferative and anti-angiogenic action of apigenin.

Together, our results indicate that apigenin prevents oncogenic UVB action in the skin, including proliferation and angiogenesis, through activation of CHK2 and possibly JNK-1, causing HuR translocation to the cytoplasm, where it binds TSP1 mRNA and increases its translational activity. In addition, TSP1 inhibits Cox-2 and poses additional restrictions on dermal angiogenesis and proliferation (Fig. 7).

## DISCUSSION

Long-term sun exposure is widespread and significantly increases the risk of epithelial cell cancers; even a single UVB exposure causes skin alterations including erythema, epidermal hyperplasia, vascular dilation and hyperpermeability [47]. Vascular changes due to UVB are especially prominent in the skin and have been attributed to the increased pro-angiogenic cytokines and chemokines, which include VEGF, basic fibroblast growth factor (bFGF), and interleukin-8 (reviewed in [48]). On the other hand, neoangiogenesis and vascular leakage are tightly controlled by TSP1, an endogenous angiogenesis inhibitor expressed at high levels by epidermal keratinocytes. Previous studies showed that acute vascular reactions to UVB are more pronounced in the skin of TSP1-null mice [7, 8]. Moreover, mice with TSP1 overexpression targeted to dermal keratinocytes exhibit weakened vascular responses to UVB irradiation, including cutaneous angiogenesis, increased endothelial cell proliferation, and apoptosis [49]. Furthermore, TSP1 transgene significantly mitigates the longer-term UVB damage in the skin, such as photo-damage and wrinkling [49]. These results stress the importance of the cutaneous vasculature in UVB-induced skin damage. They point to anti-angiogenesis as a target in preventing chronic photo-damage and skin carcinogenesis and suggest TSP1 as a means for skin cancer prevention and therapy.

Apigenin is a natural chemopreventive agent with clear anti-angiogenic effects [23, 33]. Studies in cancer cell lines linked its anti-angiogenic potential to the decreased HIF-1 or STAT-3 signaling and resultant down-regulation of pro-angiogenic VEGF [23, 50]. Other studies show that apigenin decreases production and activity of proteases including MMP-1, uPA and MMP-2 and thus attenuates angiogenesis [34]. Our recent findings in prostate cancer cells and epidermal keratinocytes demonstrate the inhibition of Cox-2 and PGE_2_ synthesis by apigenin, which could potentially hinder angiogenesis [20, 21, 39].

Here, we show for the first time that apigenin inhibits UVB-induced cutaneous angiogenesis by maintaining normal high levels of endogenous TSP1. As was shown previously, UVB irradiation decreases TSP1 protein and mRNA in cultured mouse and human keratinocytes and in mouse skin. Importantly, we showed that pre-treatment with apigenin restored high TSP1 protein levels *in vitro* and *in vivo*. Moreover, in our study apigenin strongly attenuated neoangiogenesis, proliferation and epidermal thickening in mice exposed to UVB irradiation. Furthermore, TSP1 bioactive peptide mimetic, ABT-898, restored normal angiogenesis and skin thickness and reduced proliferation in the UVB-irradiated mouse skin, suggesting that TSP1 induction by apigenin is sufficient to alleviate the acute UVB damage. Full-length TSP-1 is a large molecule with multiple functional domains (Type-2 repeats, EGF-like domain, and N-terminal region) whose effects are distinct from these of the anti-angiogenic TSR represented by ABT-898. Because it reproduces a large portion of apigenin's effects in UVB-irradiated skin and keratinocytes, we conclude that the main effects of TSP1 in this setting are mediated trough its Type I (TSR) repeats.

In contrast with apigenin, TSP1 has no documented effects on HIF-1α. However, TSP1 peptide ABT-898 was shown to decrease VEGF production in mice with orthotopic ovarian cancers [51]. Here we show, the same TSP1 peptide mimetic decreased HIF-1α levels and inhibited VEGF production in the epidermal keratinocytes caused by UVB exposure. Moreover, the same peptide reproduced another important effect of apigenin in UVB-treated cells, the reduction of Cox-2 expression. Apparently apigenin controls Cox-2 via HuR, as was shown previously [20], and this pathway involves TSP1. Cox-2 is one of the mediators of VEGF-induced angiogenesis [52]. Thus the inhibition of Cox-2 induction could be a consequence of VEGF repression by ABT-898.

Interestingly, apigenin had opposing effects on the two modifiers of angiogenesis, Cox-2 and TSP1. We have recently discovered that apigenin causes cytoplasmic localization of the RNA-binding protein HuR in UVB-treated keratinocytes where it binds the AU-rich motif in the 3′ untranslated region (UTR) of the Cox-2 mRNA and engages another RNA-binding protein, TIAR, causing translational repression [20]. Here, we found that similar to Cox-2, apigenin increased the binding of HuR to the TSP1 mRNA. However, unlike Cox-2, HuR binding increased *de novo* TSP1 synthesis. This divergent effect could be due to the differences in the molecular landscape of the 3′UTR of the Cox-2 and TSP1 genes. Previous studies demonstrate that HuR binding to the 3′UTR and stabilization of the target mRNA can lead to multiple outcomes. In some cases, HuR partners with translational repressors, such as Smaug1 and thereby blocks protein synthesis [53]. In others, HuR interferes with Argonaut (Ago) binding and formation of RISC complexes containing miRNA, and thus lifts translational repression [54]. The miRNA response to UVB is not well understood, however several miRNA have been implicated [55]. The 3′UTR of human and mouse TSP1, unlike that of Cox-2, contains multiple binding sites for the UVB-responsive miRNA including miR-141, miR-125 and miR-495, adjacent to the putative HuR binding site(s) (Supplementary Tables 1a, b and 2a, b). Thus it is possible that inhibition of RISC underlies the increased translation of the TSP1 message via apigenin - HuR axis in the UVB-treated keratinocytes and skin. Our results also indicate that Chk2 (MAPKAPK2) and possibly JNK-1 are likely mediators of HuR cytoplasmic translocation due to apigenin exposure. Interestingly, blocking JNK and Chk2 resulted in characteristic punctate patterns of HuR staining. Similar patterns were noted previously in HeLa cells subjected to heat shock and ascribed to stress granule assembly [56]. Moreover, similar granules were identified as the site where HuR target mRNAs are degraded [57].

Previous studies identify TSP1 as a p53 target in some tissues, including fibroblasts and epithelial cells of the mammary gland [58–60]. However, the mechanisms that govern TSP1 expression are tissue-specific: distinct regulatory pathways have been identified in fibroblasts and epithelial cells [61]. Moreover, p53 regulation of TSP1 may be indirect, as in colon cancer, where p53 inhibits TSP1 expression through miR-194 [62]. Similar mechanisms may be at work in cutaneous keratinocytes, where of TSP1 levels rapidly decline despite stabilization and accumulation of p53 that occurs in response to UVB-induced DNA damage.

In conclusion, our results demonstrate a previously unknown effect of apigenin in the skin, where it counters UVB-induced angiogenesis by maintaining normal high levels of the endogenous angiogenesis inhibitor, TSP1. This finding has important therapeutic implications. Apigenin is orally bioavailable and non-toxic [63] and thus presents a cheap and effective alternative to the bioactive peptides, which remain the only available type of TSP1-derived therapy [29]. This is particularly relevant because of the general importance of TSP1 in blocking acute and long-term UVB skin damage and carcinogenesis [7–9, 64–66]. TSP1 inhibits angiogenic activity by multiple growth factors [67, 68], which is especially important because in addition to VEGF, UVB induces multiple pro-angiogenic cytokines including IL-6, IL-8 and bFGF [48]. Thus maintaining sufficient levels of endogenous TSP1 is more likely to have profound effect on angiogenesis than VEGF inhibitors alone.

Our study also yields important mechanistic insight into context-dependent dichotomy of translational regulation by the RNA-binding protein HuR in the skin. We show that cytoplasmic translocation of HuR caused by apigenin and subsequent binding to the 3′ regions on the target mRNA can cause either repression or activation of translation, as is the case for Cox-2 and TSP1, respectively, the events that culminate in the inhibition of angiogenesis and proliferation in the UVB-irradiated skin.

## MATERIALS AND METHODS

### Cells and reagents

All chemicals were from Sigma–Aldrich Co (St. Louis, MO) unless otherwise specified. For detailed procedures and antibodes, see Supplementary materials section Supplementary Methods and Supplementary Table 3. The mouse keratinocyte cell line 308 was derived from Balb/c mouse skin; primary normal human epidermal keratinocytes (NHEKs) were isolated at the Skin Disease Research Center Core (Northwestern University) as described previously [69]. Specific protein kinase inhibitors for MEK1 (U0126) in the Erk1/2 pathway and Chk2 (Chk2II) were from Millipore (Billerica, MA) and used at 10 μM working concentration. Inhibitors of p38 (SB203580) and JNK-1 kinases (SP600125) from InvivoGen (San Diego, CA) were used at 10 and 50 μM, respectively.

### Treatment of cells

The cells were irradiated with FS40T12 lamps (National Biological, Twinsburg, OH, emission peak 313 nm) with Kodacel filter (Eastman Kodak, Rochester, NY) to eliminate UVC. Apigenin stock (50 mM in dimethyl sulfoxide, DMSO) was diluted in cell media. ABT-898 (Ac-GV-DalloIle-SQIRP-ethylamide), an octapeptide derived from TSP1 active internal peptide GVITRIR [30], was made to order (CPC Scientific, Sunnyvale, CA), reconstituted in water to 10 mg/ml and diluted in cell media as desired.

### Treatment of mice

We have performed only the procedures approved by Northwestern University Animal Care and Use Committee, in strict adherence to the National Institutes of Health guidelines. Six to 8-week old female SKH-1 hairless mice (Charles River, Wilmington, MA) in groups of five were treated with sham or UVB radiation (1300 J/m^2^ daily, 5 days), topical Apigenin (5 μMol) in DMSO/acetone (1:9 v/v) and subcutaneous ABT-898 (100 ng/ml in PBS, 30 mg/kg) were given 1-2 h prior to each radiation. Mice were sacrificed at 12, 24 and 48 h after final UVB exposure to harvest dorsal skins.

### Immunostaining

Immunostaining was performed as described elsewhere [70]. Stainings for CD31, Ki-67, HIF-1α and VEGF were performed at Northwestern University Mouse Histology and Phenotyping Core.

### Immunofluorescence

The cells were grown on chamber slides (Nalgene Nunc International), fixed in paraformaldehyde and permeabilized with 0.1% Triton X-100. After blocking 1h in 5% goat serum the slides were incubated overnight with HuR antibody in 5% goat serum at 4°C followed by fluorescence-labeled secondary antibodies (2h at room temperature). The cells were counterstained with diaminophenylindole (DAPI).

### Immunoblotting

The cells lysates were resolved on SDS PAGE and protein transferred onto nitrocellulose membranes, which were blocked in 5% dry milk in Tris buffered saline with 0.1% Tween 20 and incubated overnight with primary antibodies at 4°C followed by secondary antibodies (2 h, room temperature). The membranes were developed with Enhanced Chemoluminescence reagent (Amersham, Piscataway, NJ) and the signal detected by autoradiography.

### RNA isolation

Total RNA was extracted using TRIzol reagent (Life Technologies, Carlsbad, CA), following manufacturer's instructions.

### RNA interference

For HuR knockdown, siRNA duplex targeting mouse HuR and non-silencing control (Santa Cruz, Santa Cruz, CA) were introduced using Lipofectamine RNAiMAX reagent (Life Technologies, Carlsbad, CA), following manufacturer's instructions.

### Immunoprecipitation and analysis of mRNP complexes

Cytoplasmic extracts collected as described [20] were incubated 2 h on ice with HuR antibody or control IgG (15 μg/sample) followed by slow rocking with Protein A/G Plus agarose beads (30 μL/sample, 1.5 h at 4°C). The beads were precipitated by centrifugation (2000g, 2 min at 4°C), and RNA extracted using TRIzol reagent. The RNA was treated with DNAse I (DNA-free kit, Ambion, Austin, TX) and reverse transcribed using SuperScript III with random hexamer primers (Life Technologies, Carlsbad, CA). Real-time PCR was performed using TaqMan Gene Express with TSP1 primers (Applied Biosystems, Foster City, CA) in ABI Prism 7900HT system, the product normalized to GAPDH and calculations performed using ΔΔCt method.

### Analysis of Nascent TSP1 Protein

For metabolic labeling, 308 keratinocytes were pre-incubated 1 h in methionine and cysteine free DMEM (Sigma). Twelve h post-treatment, the cells were given a 15 min pulse of with 500 μCi [^35^S]L-methionine and [^35^S]L-cysteine (GE Healthcare, Piscataway, NJ) and harvested in lysis buffer as described previously [20]. Immunoprecipitation with anti-TSP1 or control antibody was carried out overnight at 4°C. Following extensive washes in 50 mM Tris-HCl (pH 7.5), 250 mM NaCl, 5 mM EDTA and 0.5% NP-40, the immunoprecipitates were resolved by SDS-PAGE and assessed by autoradiography.

### Quantitative and statistical analyses

**1. Measurements of epidermal thickness:** the number of cell layers in the epidermis was determined in H&E-stained slides by counting the number of nucleated epithelial cells along a vertical line from the stratum basale to the stratum granulosum, away from hair follicles, in 10 representative fields at 400x magnification. The epidermal thickness (μm) was similarly measured in the same areas and included the full thickness from the stratum basale to the stratum corneum.

**2. Microvascular density and Ki-67 positive nuclei** were quantified using “Object Count” function of the ImageJ64 software (National Institutes of Health). CD31-positive structures (reported as “capillaries per area”) or Ki-67 positive nuclei in the epidermis, outside of the hair follicles, were quantified in at least 10 random fields, on three sections from three individual animals. The numbers of Ki67 positive nuclei was calculated per 100 linear μm of epidermal layer and the number of CD31-positive vascular structures per 100 μm^2^ of epidermal area.

**3. Statistical significance** was calculated by pairwise comparison, with 2-tailed Student's T-test, and by one-way analysis of variance (ANOVA, Graph Pad Prism). P values below 0.05 were set as significant.

## SUPPLEMENTARY METHODS, FIGURES AND TABLES



## ABBREVIATIONS

UVB,: ultraviolet B;
COX-2,: cyclooxygenase 2;
VEGF,: Vascular Endothelial Growth Factor;
NO,: nitric oxide;
TSP1,: thrombospondin 1;
PGE_2_,: prostaglandin E_2_;
NHEKs,: normal human epidermal keratinocytes
