# Supplementary material for: Chemopreventive apigenin controls UVB-induced cutaneous proliferation and angiogenesis through HuR and thrombospondin-1
Source: Oncotarget. 2014 Oct 15;5(22):11413–27. doi: 10.18632/oncotarget.2551 (PMC4294383; doi:10.18632/oncotarget.2551)
Supplement: Supplementary file 1 [file oncotarget-05-11413-s001.pdf]

SUPPLEMENTARY METHODS, FIGURES AND TABLES

Cells and reagents

The mouse keratinocyte cell line 308 was derived from Balb/c mouse skin initiated with dimethylbenz[a]anthracene and, maintained in Suspension Minimum Essential Medium (United States Biological, Swampscott, MA) supplemented with 8% chelexed FBS (Bio-Rad Laboratories, Hercules, CA) and 0.05 mM Ca^{2+} and treated at 80% confluence. Primary normal human epidermal keratinocytes (NHEKs) were used at passages below 4.

Treatment of cells

Cultured cells were treated at 80–90% confluence. For UV exposure, the culture medium was removed and saved. The cells were rinsed with phosphate-buffered saline (PBS), irradiated, and the medium subsequently replaced. We used FS40T12 lamps (National Biological, Twinsburg, OH) with emission peak at 313 nm, in combination with Kodacel filter (Eastman Kodak, Rochester, NY) to eliminate UVC (< 295 nM). Apigenin (Sigma, St. Louis, MO) stock solutions were prepared in dimethyl sulfoxide (DMSO) and added to the culture medium to desired final concentration. The concentration of DMSO in cell cultures was less than 0.1%. ABT-898, a substituted octapeptide based on the internal active TSP1 peptide GVITRIR (Garside et al., 2010) was made to order using previously published sequence Ac-GV-DalloIle-SQIRP-ethylamide CPG Pharmaceuticlas, (CPC Scientific, Sunnyvale, CA). The peptide was reconstituted at 10 mg/ml in water and diluted in culture medium as desired.

Treatment of mice

All procedures have been approved by Northwestern University Animal Care and Use Committee and performed in accordance with the guidelines established by the National Institutes of Health. Adult (6–8 week old) female SKH-1 hairless mice (Charles River Laboratories, Wilmington, MA, USA) were assigned to groups (n=5). Mice in control group were topically treated with 0.2 ml DMSO/acetone (1:9) and treated with sham radiation. We applied UVB radiation at 1300 J/m^2 daily for 5 days. Apigenin (5 µMol) was administered topically in DMSO/acetone and ABT-898 (100 ng/ml) injected subcutaneously, 1–2 h prior to sham or UVB radiation. Mice were sacrificed to harvest dorsal skin at 12, 24 and 48 h after final UVB exposure. Dorsal skin was processed snap-frozen or formalin-fixed and processed for immunohistohemical analyses.

Immunostaining

For TSP1 and CD36, we used 5 µm-thick formalin-fixed, paraffin embedded tissue sections deparaffinized in xylene (3 × 5 min), 100% ethanol (5 min), 3% H_2O_2 in methanol (30 min), 100% ethanol (2 × 3 min) and 95% ethanol (5 min). After antigen retrieval, the slides were treated with 0.01% Triton X-100 in PBS, rinsed 3 times in PBS. For further procedures, we used the solutions and reagents from Vectastain Universal Elite ABC Kit (Biogenex, Fremont, CA) following manufacturer's instructions. The slides were blocked, incubated with primary antibodies in blocking solution (30 min each) rinsed in PBS and incubated with biotinylated secondary antibodies (30 min). The slides were then incubated with ABC reagent (30 min) rinsed in PBS and developed individually with diaminobenzydine (DAB) using distilled water to terminate the reaction. All slides were counterstained with hematoxylin. CD31 and Ki-67 stainings were performed at Northwestern University Pathology Core.

Immunofluorescence

The cells were grown on Lab-Tek Chamber Slides (Nalgene Nunc International), treated as desired, rinsed in PBS and fixed 30 min in 4% paraformaldehyde in PBS at room temperature. The cells were rinsed in PBS and treated 10 min with 0.1% Triton X-100 in PBS. After 1 h blocking (0.1% Tween20, 5% goat serum in PBS) the slides were incubated with HuR antibody in 5% goat serum overnight at 4°C. The staining was completed using goat anti-mouse IgG tagged with Alexa Fluor 488 (Invitrogen) at room temperature for 2h. The slides were mounted in Vectashield mounting medium supplemented with diaminophenylindole (DAPI).

Immunoblotting

The cells were harvested in the lysis buffer (20 mM Tris HCl, pH7.5, 150 mM NaCl, 2 mMol EDTA, 10% glycerol, 1% Triton X-100, 1 mM PMSF and protease inhibitor cocktail). Protein concentration was measured with BCA reagent (Pierce, Rockford, IL) and lysates resolved using 12% SDS PAGE and protein transferred onto nitrocellulose membranes. The membranes were blocked for 2 h at room temperature in 5% dry milk in Tris buffered saline with 0.1% Tween 20 and incubated overnight with primary antibodies. After secondary antibodies, the membranes were developed in Enhanced Chemoluminiscence reagent (Amersham, Piscataway, NJ) and the signal detected by autoradiography.

RNA interference

For HuR knockdown, siRNA duplex targeting mouse HuR and non-silencing control (Santa Cruz, Santa Cruz, CA) were introduced into 308 cells using Lipofectamine RNAiMAX reagent (Life Technologies, Carlsbad, CA), following manufacturer's instructions. After 96 hrs, cells were further subjected to UVB and/or apigenin treatment.

Immunoprecipitation of mRNP complexes and qRT-PCR

Cytoplasmic extracts collected as described previously (Tong et al. 2007) were incubated on ice with HuR antibody (15 µg/sample) or normal mouse IgG. After 2 h Protein A/G Plus agarose beads (30 µL/sample, Santa Cruz) were added and incubated with slow rocking for additional 1.5 h at 4°C. The beads with the immune complexes were precipitated by centrifugation (2000 × g, 2 min at 4°C), washed 4x with cold buffer A with protease and RNAse inhibitors, and RNA was extracted using TRIzol reagent (Life Technologies, Carlsbad, CA) as described above.

Total RNA was further subjected to DNAse I treatment (DNA-free kit, Ambion, Austin, TX) and reverse transcription using SuperScript III for the 1st strand synthesis with random hexamer primers (Life Technologies, Carlsbad, CA). Real-time PCR was performed using TaqMan Gene Express Assay (Applied Biosystems, Foster City, CA. Assay ID: Mm01335418_m1) specific for mouse TSP1 gene. Fluorescence was detected with an ABI Prism 7900HT real-time PCR system and normalized to GAPDH gene. Relative cDNA amounts were calculated using ΔΔCt method.

Analysis of Nascent TSP1 Protein

For metabolic labeling, mouse 308 keratinocyte cells were incubated for 1 h in DMEM medium without methionine and cysteine (Sigma) before treatment (UVB, with or without apigenin). At 12 h post-treatment each sample was labeled with 500 µCi L-[^{35}S]methionine and L-[^{35}S]cysteine (GE Healthcare, Piscataway, NJ) for 15 min, cells were harvested in lysis buffer as whole-cell extracts and immunoprecipitation (IP) with anti-TSP1 antibody or normal mouse IgG was carried out overnight at 4°C. Following extensive washes in washing buffer (50 mM Tris-HCl (pH 7.5), 250 mM NaCl, 5 mM EDTA and 0.5% NP-40), the immunoprecipitated materials were resolved by SDS-PAGE and assessed by autoradiography.

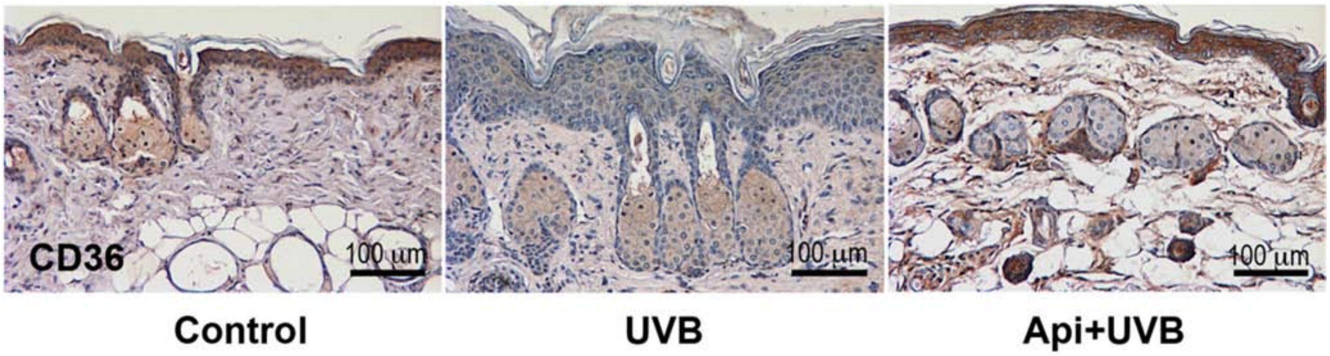

Supplementary Figure S1: SKH-1 mice were treated with UVB (5 days, 1300 J/m^2 daily). Where indicated, the animals were treated with topical apigenin (Api, 5 µMol in DMSO/acetone). Dorsal skins were harvested at 48 hr, fixed in formalin and paraffin-embedded. Five µm sections were stained with CD36 antibody.

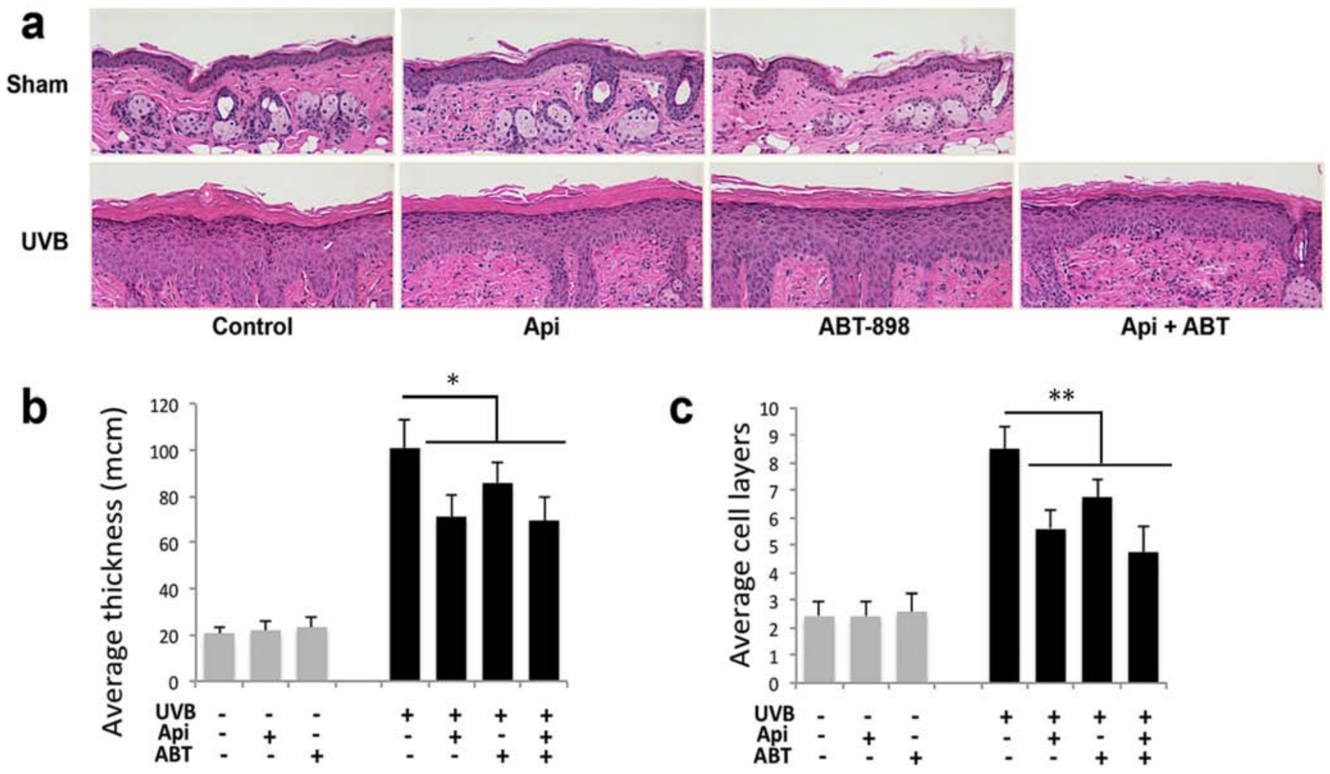

Supplementary Figure S2: Epidermal thickness at 48 hrs post UVB irradiation. SKH-1 mice were treated with UVB (5 days, 1300 J/m^2 daily). Where indicated, the animals were treated with topical apigenin (Api, 5 µMol in DMSO/acetone) and subcutaneous injections of ABT-898 (30 mg/kg). Dorsal skins were harvested at 48 hr, fixed in formalin and paraffin-embedded. Five µm sections were stained with Hematoxylin and Eosin for morphometric analysis. **(a)** H&E staining of skin sections. Note the visibly decreased thickness of the UVB-irradiated epidermis in mice treated with apigenin and/or ABT-898 **(b, c)** Measurement of epidermal thickness and keratinocyte layers. A minimum of 3 sections per animal, 3 animals for each data point was evaluated. P values are calculated using one-way ANOVA. *, P<0.01; **, P,0.006

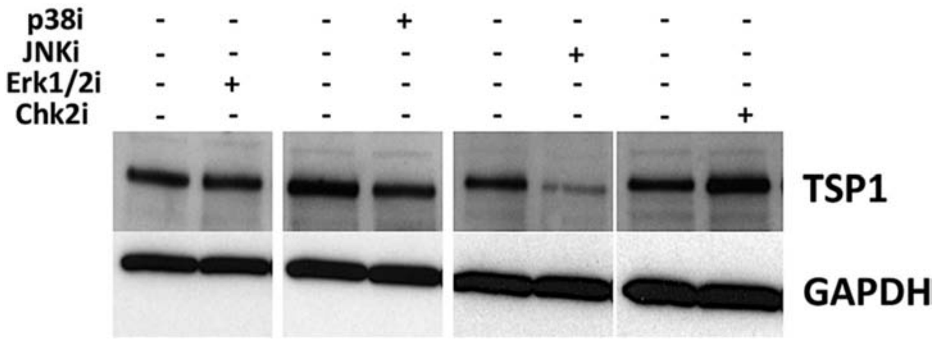

Supplementary Figure S3: The effect of MAPK and Chk2 inhibitors on basal TSP1 expression. 308 keratinocytes were treated for 12 h with U0126, SP600125, SB203580 and CHK2 inhibitor II, which inhibit p38, JNK-1, Erk1/2, and Chk2, respectively. TSP1 was detected in whole cell extracts by Western blotting and the blots re-probed with GAPDH to ascertain equal loading.

Supplementary Table S1a. **miRNA and HuR binding sites in human THBS-1 3′UTR**

miR-18a<br>miR-421 miR-383 miR-338–3p miR-18b<br>1 UCAUCAAAUUGUUGAUUGAAAGACUGAUCAUAAACCAAUGCUGGUAUUGCACCUUCUGGAACUAUGGGCUUGAGA 75
76 AAACCCCCAGGAUCACUUCUCCUUGGCUUCCUUCUUUUCUGUGCUUGCAUCAGUGUGGACUCCUAGAACGUGCGA 150
miR-217 miR-485-5p<br>151 CCUGCCUCAAGAAAAUGCAGUUUUCAAAAACAGACUCAGCAUUCAGCCUCCAAUGAAUAAGACAUCUUCCAAGCA 225
226 UAUAAACAAUUGCUUUGGUUUCCUUUUGAAAAAGCAUCUACUUGCUUCAGUUGGGAAGGUGCCCAUUCCACUCUG 300
301 CCUUUGUCACAGAGCAGGGUGCUAUUGUGAGGCCAUCUCUGAGCAGUGGACUCAAAAGCAUUUUCAGGCAUGUCA 375
376 GAGAAGGGAGGACUCACUAGAAUUAGCAAACAAAACCACCCUGACAUCCUCCUUCAGGAACACGGGGAGCAGAGG 450
miR-136 miR-194<br>451 CCAAAGCACUAAGGGGAGGGCGCAUACCCGAGACGAUUGUAUGAAGAAAAUAUGGAGGAACUGUUACAUGUUCGG 525
526 UACUAAGUCAUUUUCAGGGGAUUGAAAGACUAUUGCUGGAUUUCAUGAUGCUGACUGGCGUUAGCUGAUUAACCC 600
601 AUGUAAAUAGGCACUUAAAUAGAAGCAGGAAAGGGAGACAAAGACUGGCUUCUGGACUUCCUCCCUGAUCCCCAC 675
miR-491-5p miR-125a-3p<br>676 CCUUACUCAUCACCUGCAGUGGCCAGAAUUAGGGAAUCAGAAUCAAACCAGUGUAAGGCAGUGCUGGCUGCCAUU 750
miR-222 miR-222<br>miR-221 miR-221<br>751 GCCUGGUCACAUUGAAAUUGGUGGCUUCAUUCUAGAUGUAGCUUGUGCAGAUGUAGCAGGAAAAUAGGAAAACCU 825
miR-138 miR-410<br>826 ACCAUCUCAGUGAGCACCAGCUGCCUCCCAAAGGAGGGGCAGCCGUGCUUAUAUUUUUAUGGUUACAAUGGCACA 900
miR-613<br>miR-1<br>miR-590-3p miR-206 miR-873<br>901 AAAUUAUUAUCAACCUAACUAAAACAUUCCUUUUCUCUUUUUUCCUGAAUUAUCAUGGAGUUUUCUAAUUCUCUC 975
miR-181a-d miR-487b<br>976 UUUUGGAAUGUAGAUUUUUUUUAAAUGCUUUACGAUGUAAAAUAUUUAUUUUUUACUUAUUCUGGAAGAUCUGGC 1050
1051 UGAAGGAUUAUUCAUGGAACAGGAAGAAGCGUAAAGACUAUCCAUGUCAUCUUUGUUGAGAGUCUUCGUGACUGU 1125
miR-371-5p<br>1126 AAGAUUGUAAAUACAGAUUAUUUAUUAACUCUGUUCUGCCUGGAAAUUUAGGCUUCAUACGGAAAGUGUUUGAGA 1200
miR-590-5p<br>miR-505 miR-21<br>1201 GCAAGUAGUUGACAUUUAUCAGCAAAUCUCUUGCAAGAACAGCACAAGGAAAAUCAGUCUAAUAAGCUGCUCUGC 1275
miR-186<br>1276 CCCUUGUGCUCAGAGUGGAUGUUAUGGGAUUCUUUUUUUCUCUGUUUUAUCUUUUCAAGUGGAAUUAGUUGGUUA 1350
miR-135a-b miR-144<br>1351 UCCAUUUGCAAAUGUUUUAAAUUGCAAAGAAAGCCAUGAGGUCUUCAAUACUGUUUUACCCCAUCCCUUGUGCAU 1425
1426 AUUUCCAGGGAGAAGGAAAGCAUAUACACUUUUUUCUUUCAUUUUUCCAAAAGAGAAAAAAAUGACAAAAGGUGA 1500
miR-202<br>let-7a-f, i<br>miR-98<br>let-7g miR-377<br>1501 AACUUACAUACAAAUAUUACCUCAUUUGUUGUGUGACUGAGUAAAGAAUUUUUGGAUCAAGCGGAAAGAGUUUAA 1575
miR-141<br>miR-139-5p miR-200a<br>1576 GUGUCUAACAAACUUAAAGCUACUGUAGUACCUAAAAAGUCAGUGUUGUACAUAGCAUAAAAACUCUGCAGAGAA 1650
1651 GUAUUCCCAAUAAGGAAAUAGCAUUGAAAUGUUAAAUACAAUUUCUGAAAGUUAUGUUUUUUUUCUAUCAUCUGG 1725
miR-539<br>1726 UAUACCAUUGCUUUAUUUUUAUAAAUUAUUUUCUCAUUGCCAUUGGAAUAGAUAUCUCAGAUUGUGUAGAUAUGC 1800

(*Continued*)

	miR-340 miR-19a<br>miR-410 miR-19b	
1801	UAUUUAAAUAAUUUAUCAGGAAAUACUGCCUGUAGAGUUAGUAUUUCUAUUUUUAUAUAAUGUUUGCACACUGAA	1875
	miR-186 miR-495	
1876	UUGAAGAAUUGUUGGUUUUUUCUUUUUUUUGUUUUGUUUUUUUUUUUUUUUUUUUUUUGCUUUUGACCUCCCAUUU	1950
	miR-182 miR-384	
1951	UUACUAUUUGCCAAUACCUUUUUCUAGGAAUGUGCUUUUUUUUGUACACAUUUUUAUCCAUUUUACAUUCUAAAG	2025
	miR-494<br>miR-381 miR-144 miR-375	
2026	CAGUGUAAGUUGUAUAUUACUGUUUCUUAUGUACAAGGAACAACAAUAAAUCAUAUGGAAAUUUAUAUUUAU	2097

Note potential HuR binding sites (highlighted in blue) and binding sites for UVB-regulated miRNA (highlighted in yellow)

Supplementary Table S1b. miRNA and HuR binding sites in mouse THBS-1 3′UTR

1	miR-18a<br>miR-18b<br>miR-33	UCAUCAGCUGCCAAUCAUAACCAGCGCUGGCAAUGCACCUUCUAAAAACAAGGGCUAGAGAAACCCCCCACCCCU	75
76		GCCGGGAUCGCCUUUCCUCGCCUUCCUUGCCUCUCUUCUUGCAUAGUGUGGACUUGUAAAGCCUGAGACCUGCCU	150
151	miR-326<br>miR-217<br>miR-330	CAAGAAAAUGCAGUUUUCAAACCCAGAGUCAGCACUCGGCCUUUAACGAAUGAGAAUGCAUCUUCCAAGACCAUG	225
226		AAGAGUUCCUUGGGUUUGCUUUUGGGAAAGCCAAAGCGCCUAUUUACUUCCCACUAGGAAGGUGCCCGCUCCACU	300
301	miR-149	CUGCCUUACUCACAGAGCCAGAACUUCUUCGAGGCCACCUCUGAGCAGCACACACAGAAGCAUUUUCAGGCAUGU	375
376		CAAAGAAAGGAAAAAAUGACUCACUAGAACUCACCGCCAAACAACCUCUGACAUAGGUCCUGAGAUGUGGGGAGG	450
451		CAGGAGCCAAAGCUCUAGGGAGGGCAUGUACCCAAGAGAUGACUGUAUGAAAAUGUGGAGGAGCUGUUCGGUACU	525
526	miR-15a, b<br>miR-497<br>miR-16<br>miR-195<br>miR-322<br>miR-103<br>miR-107	AAAUCAUUUUCAGGGGACAGACAGACUUGCUGCAUUUCUGCAUGCUGCUGGUGAGAGCUGAUUGACCCAAUCUUC	600
601		CACACAGGCACUUGAGCAAGCAGGGAAGGGAGGGAGAUCAUAGCUUCUGGACUUUCUCCCUUUGGGCACUUCUCA	675
676	miR-125a-3p	CCUGCAGUGGCCAGGGUAGGGGUCAGAAGUGUGGGCCAUGCUGGCUGCCCUUGACUGGUCACGCUGAAACUGUUA	750

Note potential HuR binding sites (highlighted in blue) and binding sites for UVB-regulated miRNA (highlighted in yellow)

Supplementary Table S2a. miRNA and HuR binding sites in human Cox-2 3′UTR

1 AAGUCUAAUGAUCAUAUUUAUUUAUUUAUAUGAACCAUGUCUAUUAAUUUAAUUAUUUAAUAAUAUUUAUAUUAA 75
76 ACUCCUUAUGUUACUUAACAUCUUCUGUAACAGAAGUCAGUACUCCUGUUGCGGAGAAAGGAGUCAUACUUGUGA 150
151 AGACUUUUAUGUCACUACUCUAAAGAUUUUGCUGUUGCUGUUAAGUUUGGAAAACAGUUUUUAUUCUGUUUUAUA 225
miR-1297<br>miR-26a, b miR-374a, b<br>226 AACCAGAGAGAAAUGAGUUUUGACGUCUUUUUACUUGAAUUUCAACUUAUAUUAUAAGAACGAAAGUAAAGAUGU 300
miR-542-3p miR-33a<br>miR-758 miR-33b<br>301 UUGAAUACUUAAACACUGUCACAAGAUGGCAAAAUGCUGAAAGUUUUUACACUGUCGAUGUUUCCAAUGCAUCUU 375
376 CCAUGAUGCAUUAGAAGUAACUAAUGUUUGAAAUUUUAAAGUACUUUUGGUUAUUUUUCUGUCAUCAAACAAAAA 450
miR-411<br>451 CAGGUAUCAGUGCAUUAUUAAAUGAAUAUUUAAAUUAGACAUUACCAGUAAUUUCAUGUCUACUUUUUAAAAUCA 525
miR-125a-3p<br>526 GCAAUGAAACAAUAAUUUGAAAUUUCUAAAUUCAUAGGGUAGAAUCACCUGUAAAAGCUUGUUUGAUUUCUUAAA 600
601 GUUAUUAAACUUGUACAUAUACCAAAAAGAAGCUGUCUUGGAUUUAAAUCUGUAAAAUCAGUAGAAAUUUUACUA 675
676 CAAUUGCUUGUUAAAAUAUUUUAUAAGUGAUGUUCCUUUUUCACCAAGAGUAUAAACCUUUUUAGUGUGACUGUU 750
miR-132 miR-141<br>miR-212 miR-200a<br>751 AAAACUUCCUUUUAAAUCAAAAUGCCAAAUUUAUUAAGGUGGUGGAGCCACUGCAGUGUUAUCUUAAAAUAAGAA 825
826 UAUUUUGUUGAGAUAUUCCAGAAUUUGUUUAUAUGGCUGGUAACAUGUAAAAUCUAUAUCAGCAAAAGGGUCUAC 900
miR-379 miR-137<br>901 CUUUAAAAUAAGCAAUAACAAAGAAGAAAACCAAAUUAUUGUUCAAAUUUAGGUUUAAACUUUUGAAGCAAACUU 975
miR-148a,b<br>miR-152<br>976 UUUUUUAUCCUUGUGCACUGCAGGCCUGGUACUCAGAUUUUGCUAUGAGGUUAAUGAAGUACCAAGCUGUGCUUG 1050
1051 AAUAAUGAUAUGUUUUCUCAGAUUUUCUGUUGUACAGUUUAAUUUAGCAGUCCAUAUCACAUUGCAAAAGUAGCA 1125
miR-202 miR-203<br>1126 AUGACCUCAUAAAAUACCUCUUCAAAAUGCUUAAAUUCAUUUCACACAUUAAUUUUAUCUCAGUCUUGAAGCCAA 1200
1201 UUCAGUAGGUGCAUUGGAAUCAAGCCUGGCUACCUGCAUGCUGUUCCUUUUCUUUUCUUCUUUUAGCCAUUUUGC 1275
1276 UAAGAGACACAGUCUUCUCAUCACUUCGUUUCUCCUAUUUUGUUUUACUAGUUUUAAGAUCAGAGUUCACUUUCU 1350
1351 UUGGACUCUGCCUAUAUUUUCUUACCUGAACUUUUGCAAGUUUUCAGGUAAACCUCAGCUCAGGACUGCUAUUUA 1425
miR-28-5p<br>miR-708<br>1426 GCUCCUCUUAAGAAGAUUAAAAGAGAAAAAAAAAGGCCCUUUUAAAAAUAGUAUACACUUAUUUUAAGUGAAAAG 1500
1501 CAGAGAAUUUUAUUUAUAGCUAAUUUUAGCUAUCUGUAACCAAGAUGGAUGCAAAGAGGCUAGUGCCUCAGAGAG 1575
1576 AACUGUACGGGGUUUGUGACUGGAAAAAGUUACGUUCCCAUUCUAAUUAAUGCCCUUUCUUAUUUAAAAACAAAA 1650
miR-146a, b-5p<br>miR-137<br>1651 CCAAAUGAUAUCUAAGUAGUUCUCAGCAAUAAUAAUAAUGACGAUAAUACUUCUUUUCCACAUCUCAUUGUCACU 1725
miR-101<br>miR-144<br>1726 GACAUUUAAUGGUACUGUAUAUUACUUAAUUUAUUGAAGAUUAUUAUUUAUGUCUUAUUAGGACACUAUGGUUAU 1800
miR-542-3p<br>miR-758<br>1801 AAACUGUGUUUAAGCCUACAAUCAUUGAUUUUUUUUUGUUAUGUCACAAUCAGUAUAUCUUCUUUGGGGUUACCU 1875

(*Continued*)

	miR-9	
1876	CUCUGAAUAUUAUGUAAACAAUCCAAAGAAAUGAUUGUAUUAAGAUUUGUGAAUAAAUUUUUAGAAAUCUGAUUG	1950
	miR-181a-d<br>miR-543	
1951	GCAUAUUGAGAUAUUUAAGGUUGAAUGUUUGUCCUUAGGAUAGGCCUAUGUGCUAGCCCACAAAGAAUAUUGUCU	2025
	miR-181a-d<br>miR-183	
2026	CAUUAGCCUGAAUGUGCCAUAAGACUGACCUUUUAAAAUGUUUUGAGGGAUCUGUGGAUGCUUCGUUAAUUUGUU	2100
	miR-128	
2101	CAGCCACAAUUUAUUGAGAAAAUAUUCUGUGUCAAGCACUGUGGGUUUUAAUAUUUUUAAAUCAAACGCUGAUUA	2175
	miR-200b, c miR-200c<br>miR-410	
2176	CAGAUAAUAGUAUUUAUAUAAAUAAUUGAAAAAAAUUUUCUUUUGGGAAGAGGGAGAAAAUGAAAUAAAUAUCAU	2250
	miR-543	
2251	UAAAGAUAACUCAGGAGAAUCUUCUUUACAAUUUUACGUUUAGAAUGUUUAAGGUUAAGAAAGAAAUAGUCAAUA	2325
2326	UGCUUGUAUAAAACACUGUUCACUGUUUUUUUUAAAAAAAAAACUUGAUUUGUUAUUAACAUUGAUCUGCUGACA	2400
2401	AAACCUGGGAAUUUGGGUUGUGUAUGCGAAUGUUUCAGUGCCUCAGACAAAUGUGUAUUUAACUUAUGUAAAAGA	2475
2476	UAAGUCUGGAAAUAAAUGUCUGUUUAUUUUUGUACUAUUUAAAAAUUGACAGAUCUUUUCUGAAG	2540

Note potential HuR binding sites (highlighted in blue) and the binding sites for UVB-regulated miRNA

Supplementary Table S2b. **miRNA and HuR binding sites in mouse Cox-2 3′UTR**

1	miR-411 miR-23a, b<br>AAGUCUACUGACCAUAUUUAUUUAUUUAUGUGAAGAAUUUAAUUUAAUUAUUUAAUAUUUAUACUGAAUUUUUUU	75
76	UCAUGUAACAUCUUCCAUAACAGAAGGCAAUGUUCUUGAACAAUGUUACAUUUGUGAAGAUUCCCUCCGGUGUUU	150
151	GUCCUUUAAAUAUGUGUUACCUGAAACUGAAAGGAAAUCAGCAUUCAUUCCUCUACAUAAGCCAGUGAGAAGGGA	225
226	miR-340-5p<br>miR-26a, b miR-433<br>AAUGAAUUUUGAUAUCUUUAUACUUGAAUUUCAGAUCAUGAUUAGCUUAACAAGAACCAAGGAAAAAUGUAUGAA	300
301	miR-103<br>miR-107 miR-875-5p<br>UAUGUGAGUGUUGUUACAAGAUGAAAAAUGCUGCAGGUAUCAACACUGUUGGUUACAACUGUGUCUUCUUUACUA	375
376	UGAUAGGAGCAUGUAAUGUGGAAUUCUUCUUAAAUCUUGCAUAUCUUUAUCUCAUCAAACAAAGGGGUCCAAGUU	450
451	CAGUUUUAAAUAAGCAUUUAAGGCAGAUACUGACAACAAUCUCAUUUUUUAAAAUGUUGUCUUGAGACAAAUAAU	525
526	miR-150<br>UUGAAAUUUCUAAAUUGGGAGUUUGAAUCACUUUUGAAAGCUCUUACUUUCUUAAGCUGUCAGGUUUGUACCGAC	600
601	AUGGAGUAAACAGCUAUCAUAAACGUAAAUCUCCAAAACUAGUAGAAAUUAUGUCAUGAUUGAUGGUUAAGAUAC	675
676	miR-376b<br>miR-219 miR-381<br>CAUGUCAGGGAUUGUCUUUUCUUAGAAGUAGUGAAAGCUACUUACUAUGACAAUCAGACCUUCCUUGUAUGUCAA	750
751	miR-338-3p miR-342-3p<br>AAUGCUGGUGUGGAAGGUGGUGGAGCCCGUGCUGCUCUGUCUUAACUAUGAGUGUGAGCUUUAAAGCUCGUUGAU	825
826	GAGUGGUAGCCAGCAAAGCCUAGAGCAACAAAAGCUUCUACAAAGGAACUAACCAAGAACAAAGAAGGGUUCCCA	900
901	AUUAAAGAUCACAUUCAGGGUUAAACUUCCAAAGGAGACAUCCUGAUCCUGGUUUUGUGCUGGCCUGGUACUCAG	975
976	miR-342-3p<br>UAGGUUUUUGCUGUGAGGUUAAAGACUUGCCAGGCUGAACUUCGAAACAGUUUUUCUGUUGCACAGUAUGAUGUA	1050
1051	miR-137<br>miR-33 miR-7a, b<br>ACAGUCCAUCUCUCAAUGCAAUAGGUAUCAGUGGCCUCGUGAGCUUCUUCACAAUAUUGAUAUGUCUUCCAGCCC	1125
1126	miR-195<br>miR-322<br>miR-15a, b<br>miR-497<br>miR-342-3p miR-16<br>AUUGAACCUGGACUGCAGAAGGCCCCAUGUCAUGUGUGAGCUCAGCCUGGAUGCCAGCAUUGCUGCUCCUCUUAG	1200
1201	miR-490<br>UUCCGUUUCUCGUGGUCACUUUACUACGAGAAACGCUGAUUGGGUUUUCGUAGCUGUGUUCCAGGUUUUUAGUAU	1275
1276	miR-186 miR-154 miR-26a, b<br>CAGAACUAUUCUUUCUUUAACCUCUAUUCAUAUUUUCUCUACUUGAAGUUUUACAUUCAGGAAAACCUCAGCUCA	1350
1351	miR-129-5p<br>GGACUACUAUGUACCUCCCCUUUGGAGGGAAAAAUUAUUUUAGGUAAAAGGCAAAAAUUUUUUAAAAAUAUUUUU	1425
1426	miR-340-5p miR-873<br>AUUUAUAAUUAUAUGGAAGGGCCCUACCAAGAUGCUAGAAAUAUAGGGAGUUCCUGACAAGAAAUUUCCAUUCUU	1500
1501	AUUCUGAAGAAUUGCUUUCUUACUUAAAAACAAAGACAGUUUGUGAGUAGUUCUGGGCAAUAGGGAUAAAUAUAA	1575
1576	AACAAUAAUGAUGAUCAUUUUCUACAUCUCAUUAUCAGCUGAGGUACUGUAUAUUACUGAAUUUAUUGAAGAUAG	1650
1651	UUAUGUCUUUUAGACAUUGUUGUUAUAAACUAUGUUUAAGCCUACUACAAGUGUUUCUUUUUGCAUUAUGUUGGA	1725
1726	AUUGAUGUACCUUUUUUAUGAUUACCUCUCUGAACUAUGGUGUGAACAAUCAAACAAAAUGAUGAGAUUAACGUU	1800
1801	CAUGGAUAAAUUCUAAGAAAACUAGUGUAUUUUUUUGAAAAGUUUGAAGUUAGAACUUAGGCUGUUGGAAUUUAC	1875
1876	GCAUAAAGCAGACUGCAUAGAUCCAAUAUUGACUGACCCAAGCAUGUUAUAAAGACUGACAUUUUAGACAUUUUG	1950

(Continued)

1951 AAGGCCCUGUAAGUGUUUAUUAAUUAGUUAGAACUUAAUUGAUUAAAAAAUAUAUCCAAAGCACUAUAGGCAUUA 2025	
miR-132<br>miR-212<br>miR-144 miR-410 miR-374<br>2026 GAAUUCGUGCAUCAAGAAAUGAUGACAAAUAAUACUGUUAUUUAUAUAAAUAACUAAAAAGGGUGUCUAAUGAAG 2100	
2101 AAAUAUAUUUUAUUACAAAGAAAUUAUAAAACAUUUUGAAGAUUAUAUGCUUUAAAAGUUUAAGAUGAAAAAAUA 2175	
2176 AUCAACCUUAGAAAAAAUGUAUAAAAAUAUAUAAAUUGUUAAUGUCAUUGAUUAA 2230	

Note potential HuR binding sites (highlighted in blue) and the binding sites for UVB-regulated miRNA

Supplementary Table S3. Antibodies used in the study

**Primary antibodies**				
**Antigen**	**Antibodies**	**Source**	**Dilution**	**Blocking agent**
β-actin	Rabbit IgG (I-19)	Santa Cruz (Santa Cruz, CA)	1:1000 (WB)	5% dry milk in TBS-T
CD36	Rabbit IgG	Novus (Littleton, CO)	1:100 IHC	Normal horse serum, Vectastain kit, as directed
Cox-2	Rabbit IgG (C-20)	Santa Cruz (Santa Cruz, CA)	1:1000 (WB)	5% dry milk in TBS-T
TSP1	Mouse IgG (A4.1)	Novus (Littleton, CO)	1:1000 (WB) 1:100 (IHC)	5% dry milk (WB)<br>Vectastain (IHC)
HuR	Mouse IgG (3A2)	Santa Cruz (Santa Cruz, CA)	1:1000 (WB)<br>1:100 (IF)<br>1:100 (IP)	5% dry milk (WB)<br>5% goat serum (IF)<br>N/A
HuR	Mouse IgG (19F12)	Clonegene	1:100 (IHC)	5% goat serum
α-Tubulin	Mouse IgG (B-7)	Santa Cruz (Santa Cruz, CA)	1:2000 (WB)	5% dry milk in TBS-T
**Secondary antibodies**				
Source/target	Tag	Application	Source	Dilution
Rabbit anti-mouse	HRP	WB	Jackson Immunoresearch (West Grove, PA)	1:1000
Goat anti-rabbit	HRP	WB	Jackson Immunoresearch (West Grove, PA)	1:1000
Goat anti-mouse	HRP	WB	Jackson Immunoresearch (West Grove, PA)	1:1000
Goat anti-mouse	Biotin	IHC	Jackson Immunoresearch (West Grove, PA)	1:1000
Goat anti-rabbit	Biotin	IHC	Biogenex, Fremont, CA	1:1000
Goat anti-mouse	Alexa Fluor 48	Immunofluorescence	Invitrogen	1:500
